# Possible Association between the Use of Proton Pump Inhibitors and H_2_ Receptor Antagonists, and Esophageal Cancer: A Nested Case–Control Study Using a Korean National Health Screening Cohort

**DOI:** 10.3390/ph15050517

**Published:** 2022-04-22

**Authors:** Hyo Geun Choi, Hong Kyu Lee, Ho Suk Kang, Hyun Lim, Joo-Hee Kim, Ji Hee Kim, Nan Young Kim, Seong-Jin Cho, Eun Sook Nam, Kyueng-Whan Min, Mi Jung Kwon

**Affiliations:** 1 Department of Otorhinolaryngology-Head & Neck Surgery, Hallym University Sacred Heart Hospital, Hallym University College of Medicine, Anyang 14068, Korea; pupen@naver.com; 2 Department of Thoracic and Cardiovascular Surgery, Hallym University Sacred Heart Hospital, Hallym University College of Medicine, Anyang 14068, Korea; hklee0228@hallym.or.kr; 3 Division of Gastroenterology, Department of Internal Medicine, Hallym University Sacred Heart Hospital, Hallym University College of Medicine, Anyang 14068, Korea; hskang76@hallym.or.kr (H.S.K.); hlim77@hallym.or.kr (H.L.); 4 Division of Pulmonary, Allergy, and Critical Care Medicine, Department of Medicine, Hallym University Sacred Heart Hospital, Hallym University College of Medicine, Anyang 14068, Korea; luxjhee@gmail.com; 5 Department of Neurosurgery, Hallym University Sacred Heart Hospital, Hallym University College of Medicine, Anyang 14068, Korea; kimjihee.ns@gmail.com; 6 Hallym Institute of Translational Genomics and Bioinformatics, Hallym University Medical Center, Anyang 14068, Korea; honeyny78@gmail.com; 7 Department of Pathology, Kangdong Sacred Heart Hospital, Hallym University College of Medicine, Seoul 05355, Korea; apilas@hanmail.net (S.-J.C.); esnam@kdh.or.kr (E.S.N.); 8 Department of Pathology, Hanyang University Guri Hospital, Hanyang University College of Medicine, Guri 11923, Korea; kyueng@gmail.com; 9 Department of Pathology, Hallym University Sacred Heart Hospital, Hallym University College of Medicine, Anyang 14068, Korea

**Keywords:** esophageal cancer, proton pump inhibitor, H_2_-receptor antagonist, nested case–control study, nationwide health insurance research database, mortality

## Abstract

Although safety concerns regarding proton pump inhibitor (PPI)/H2-receptor antagonists (H2RA) in the incident esophageal cancer have been raised, the Asian-based report is unclear. We investigated the estimated likelihood of incident esophageal cancer—its mortality depending on prior history of PPI/H2RA use—and gastroesophageal reflux disease (GERD) in Koreans. Using the Korean National Health Insurance Service-Health Screening Cohort data (2002–2015), a case–control study was retrospectively conducted, including 811 patients with incident esophageal cancer and 3244 controls matched with sex, age, income, and residence. Propensity score overlap weighting was adjusted to balance the baseline covariates. Overlap propensity score-weighted logistic regression analyses were assessed to determine associations of the prior exposure of PPI/H2RA (current vs. past) and the medication duration (<30-, 30–90-, vs. ≥90-days) with incident esophageal cancer and its mortality among the total participants or those with/without the GERD episodes, after adjusting for multiple covariates including PPI/H2RA. The current exposure to either PPI or H2RA showed higher odds for incident esophageal cancer than the nonuser group ([13.23; 95%CI 10.25–17.06] and [4.34; 95%CI 3.67–5.14], respectively), especially in all adults over the age of 40 years without GERD. Both current and past exposures to PPI showed a decreased probability of mortality compared with those of the nonuser group ([0.62; 95%CI 0.45–0.86] and [0.41; 95%CI 0.25–0.67], respectively). However, current or past exposure to H2RA harbored the mutually different likelihoods for mortality depending on the presence of GERD and old age. This study carefully speculates on the possible link between PPI/H2RA and incident esophageal cancer in the Korean population. Mortality appears to be affected by certain risk factors depending on drug types, exposure history, old age, and the presence of GERD.

## 1. Introduction

Esophageal carcinomas are a major global health threat with the seventh prevalence and the sixth cancer-related mortality ranks, with the incidence increasing over the past decades [1,2]. Esophageal carcinomas are one of the most aggressive therapeutically challenging malignant tumors, with a five-year survival rate of less than 15–30%, even for patients who receive curative surgery [1,2,3]. In Korea, esophageal cancers stand out as the 8th most common malignancy of cancer-related death, which is a considerably high rank compared with the low incidence of esophageal cancer in Korea [3,4]. These days, special attention is given to an increasing trend of esophageal cancer and gastroesophageal reflux disease (GERD) in Asia, including Korea [3,4]. Because of recurrent reflux of stomach contents, GERD might cause esophageal injury, inflammation, activation of proliferative signals, and ultimately DNA damage and malignant transformation. However, GERD is very common, and most people who have it would rarely develop esophageal cancer. There might be assumed to be potential links between two diseases. Since the endoscopic surveillance seems not to suppress an increment rate or mortality risk of esophageal cancer [4,5], the identification of modifiable risk factors that would enable us to develop effective prevention strategies is a fundamental priority in reversing the current rising incidence of esophageal carcinoma [6].

The risk involved in esophageal cancer may increase with old age, male sex, smoking, alcohol drinking, obesity, Barrett’s esophagus, acid peptic disorders, GERD, other malignancies, medications, environmental exposures, diet, and nutrition [1,6,7,8]. In terms of medication factors, proton pump inhibitors (PPIs) and H_2_-receptor antagonists (H2RA) are commonly prescribed acid-suppressive agents worldwide and the standard treatment for upper gastrointestinal disease [9]. Although these drugs are considered safe, there has been a safety concern that the use of acid-suppressive medication might increase the risk for esophageal cancers [10,11,12,13,14,15]. The most preferred rationale for this theory is that PPI-induced hypergastrinemia may stimulate downstream signaling and promote cell proliferation and may actually lead to implications for esophageal cancer risk [16,17]. The disclosure that ranitidine, as an H2RA, is adulterated with *N*-nitrosodimethylamine—a probable carcinogen—has raised the concern of a gastrointestinal cancer-causing effect; however, verification remains indecisive [18,19]. Experimental evidence has also supported that pantoprazole as PPI enhances tumor growth and reduces the antitumor activity of gemcitabine in fibrosarcoma-bearing immunocompetent mice [20]. However, most epidemiologic studies conducted in Western countries and several meta-analyses have denied the carcinogenic effect of PPI or H2RA use on developing esophageal cancer [7,19,21,22,23,24,25]. Rather, they claimed the preventive effect of PPI or H2RA against esophageal cancers [7,19,21,22,23,26,27], with up to 71% lower risk of neoplastic progression [23]. In vitro study has shown significant antitumor effects of PPI in diminishing invasiveness and promoting apoptosis in esophageal cancer cells [28].

Despite the growing studies, there has been little available information based on the Asian population, including Korean, on this issue. Given the widespread use of PPI and H2RA over the decades and the current increasing trend of GERD and esophageal cancers in Asia, including Korea [1,4,8], ethnicity seems to contribute to predisposition to the adverse effect of these drugs [13]. East Asians are known to have a slow metabolizer of PPIs and H2RA compared with people in other ethnic groups, as a consequence of a genetically lowered manifestation of hepatic cytochrome p450 enzyme [29], a prerequisite for the metabolism of PPIs and H2RA [30]. Only one study attested to this claim [13], finding an incremented hazard of esophageal cancer in people with prior PPI use, which has yet to be reproduced. Since that study originally did not aim to elucidate the relationship of PPI and H2RA with subsequent esophageal cancers [13], the investigators did not conduct a broad span of subgroup analysis for proceeding esophageal cancer and its mortality in conjunction with PPIs and H2RA. Concerning this vulnerability to drug metabolism in Asian people [29], validation for the safety issue requires prompt further study.

Thus, we hypothesized that PPI and H2RA use could adversely impact the development of esophageal cancers and their mortality in the Korean population with increasing episodes of GERD. To test this hypothesis, we retrospectively carried out a nested case–control study making use of nationwide public health care data to identify the relationship among prior use of PPIs or H2RA, incident esophageal cancers, and mortality depending on the GERD episodes.

## 2. Results

### 2.1. Baseline Characteristics

Overall, 811 patients with esophageal cancer and 3244 people in the comparison group were registered in this study after matching the propensity scores. The baseline characteristics between the two groups were not exactly balanced before adjustment via the overlap weighting method (Table S1). Patients with esophageal cancers were more likely to be nonobese, smokers, frequent alcohol drinkers, and more likely to have more comorbidities, more episodes of GERD, and more cumulative days using PPI or H2RA compared with control participants.

After adjusting for imbalances between the groups by overlap weighting, standardized mean differences became minimized, and the balanced baseline covariates were achieved (standardized difference = 0.00) (Table 1).

### 2.2. Associations of Prior Use of PPI and Its Duration with Esophageal Cancer

**Figure 1:**
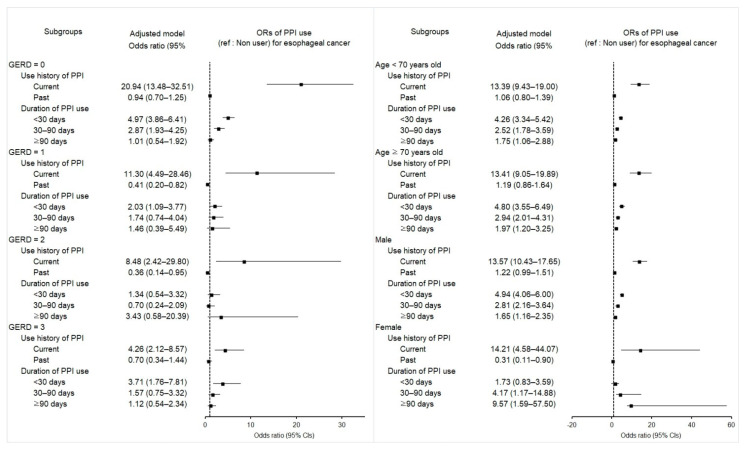
Forest plots for multivariable conditional logistic regression depicting the overlap weighted odds ratios (95% confidence intervals) of previous PPI exposure history and use duration for incident esophageal cancer according to the number of GERD episodes, age, and sex. The reference is a nonuser. Full results of the crude and adjusted overall weighted models are available in Supplementary Table S2.

We investigated the potential relevance of exposure history of PPIs with the incidence of esophageal cancer compared to the controls (Table 2). Current PPI users were associated with much higher odds for esophageal cancer than the nonuser comparison group (adjusted odds ratio [aOR] 13.23; 95% confidence interval [CI] 10.25–17.06; *p* < 0.001). This current PPI use was related to increased odds of esophageal cancer in most subgroups of patients independent of the number of GERD episodes, sex, or age, as shown in the forest plot (Figure 1 and Table S2). In contrast, past PPI use showed no statistical association with the incidence of esophageal cancer.

The odds of incident esophageal cancer remarkably increased irrespective of the overall medication days of PPIs in both crude and adjusted models (Table 3). The duration of PPI prescription in participants of <30-, 30–90-, or ≥90-days proved higher likelihood for esophageal cancer compared with those in the comparison group (4.59 [95% CI 3.81–5.53, *p* < 0.001]; 2.79 [95% CI 2.17–3.60, *p* < 0.001]; 1.80 [95% CI 1.28–2.52, *p* = 0.001], respectively). In the subgroup analyses, PPI use for <30 days remained consistently related to a high probability of carrying esophageal cancer among all ages, among those with no GERD episodes, one episode, or ≥3 episodes, and in men. The significance shown in the period of 30–90 days was consistent in the subgroups with no GERD episodes, all ages, and both sexes. All ages and both sexes were also significant subgroups that strongly maintained the relevance of PPI administration for ≥90 days to esophageal cancer.

**Table 2:** Crude and overlap propensity score weighted odds ratios of proton pump inhibitor (ref: nonuser) for esophageal cancer with subgroup analysis by the number of GERD episodes.

Characteristics	N of Esophageal Cancer	N of Control	Odds Ratios for Esophageal Cancer (95% CI)			
	Exposure/Total (%)	Exposure/Total(%)	Crude	*p*	Overlap Weighted Model †	*p*
Total participants (n = 4055)						
Exposure						
Current	279/811 (34.4)	107/3244 (3.3)	16.1 (12.61–20.56)	<0.001 *	13.23 (10.25–17.06)	<0.001 *
Past	85/811 (10.5)	376/3244 (11.6)	1.40 (1.08–1.80)	0.011 *	1.15 (0.93–1.41)	0.194
Duration of PPI use (days)						
<30	231/811 (28.5)	253/3244 (7.8)	5.64 (4.60–6.92)	<0.001 *	4.59 (3.81–5.53)	<0.001 *
30–90	83/811 (10.2)	141/3244 (4.3)	3.64 (2.72–4.85)	<0.001 *	2.79 (2.17–3.60)	<0.001 *
≥90	50/811 (6.2)	89/3244 (2.7)	3.47 (2.42–4.98)	<0.001 *	1.80 (1.28–2.52)	0.001 *
GERD = 0 (n = 3304)						
Exposure						
Current	102/507 (20.1)	38/2797 (1.4)	18.6 (12.62–27.41)	<0.001 *	20.94 (13.48–32.51)	<0.001 *
Past	30/507 (5.9)	161/2797 (5.8)	1.29 (0.86–1.93)	0.216	0.94 (0.70–1.25)	0.673
Duration of PPI use (days)						
<30	99/507 (19.5)	121/2797 (4.3)	5.67 (4.26–7.55)	<0.001 *	4.97 (3.86–6.41)	<0.001 *
30–90	26/507 (5.1)	49/2797 (1.8)	3.68 (2.26–5.99)	<0.001 *	2.87 (1.93–4.25)	<0.001 *
≥90	7/507 (1.4)	29/2797 (1.0)	1.67 (0.73–3.84)	0.226	1.01 (0.54–1.92)	0.968
GERD = 1 (n = 281)						
Exposure						
Current	56/107 (52.3)	14/174 (8.0)	10.42 (5.13–21.20)	<0.001 *	11.3 (4.49–28.46)	<0.001 *
Past	18/107 (16.8)	74/174 (42.5)	0.63 (0.33–1.22)	0.171	0.41 (0.20–0.82)	0.013 *
Duration of PPI use (days)						
<30	53/107 (49.5)	59/174 (33.9)	2.34 (1.36–4.04)	0.002 *	2.03 (1.09–3.77)	0.026 *
30–90	15/107 (14.0)	23/174 (13.2)	1.70 (0.79–3.65)	0.174	1.74 (0.74–4.04)	0.202
≥90	6 / 107 (5.6)	6/174 (3.4)	2.61 (0.78–8.66)	0.118	1.46 (0.39–5.49)	0.579
GERD = 2 (n = 174)						
Exposure						
Current	44/76 (57.9)	13/98 (13.3)	5.42 (2.35–12.46)	<0.001 *	8.48 (2.42–29.80)	0.001 *
Past	12/76 (15.8)	53/98 (54.1)	0.36 (0.16–0.84)	0.018 *	0.36 (0.14–0.95)	0.038 *
Duration of PPI use (days)						
<30	38/76 (50.0)	38/98 (38.8)	1.60 (0.78–3.28)	0.199	1.34 (0.54–3.32)	0.534
30–90	12/76 (15.8)	23/98 (23.5)	0.83 (0.34–2.04)	0.692	0.70 (0.24–2.09)	0.527
≥90	6/76 (7.9)	5/98 (5.1)	1.92 (0.52–7.13)	0.330	3.43 (0.58–20.39)	0.176
GERD ≥ 3 (n = 296)						
Exposure						
Current	77/121 (63.6)	42/175 (24.0)	4.34 (2.26–8.36)	<0.00*	4.26 (2.12–8.57)	<0.001 *
Past	25/121 (20.7)	88/175 (50.3)	0.67 (0.34–1.35)	0.265	0.70 (0.34–1.44)	0.328
Duration of PPI use (days)						
<30	41/121 (33.9)	35/175 (20.0)	2.77 (1.38–5.59)	0.00 *	3.71 (1.76–7.81)	0.001 *
30–90	30/121 (24.8)	46/175 (26.3)	1.54 (0.76–3.13)	0.228	1.57 (0.75–3.32)	0.234
≥90	31/121 (25.6)	49/175 (28.0)	1.50 (0.74–3.02)	0.258	1.12 (0.54–2.34)	0.761

Abbreviations: GERD—gastroesophageal reflux disease; N—number; 95% CI—95% confidence interval; PPI—proton pump inhibitor. * Significance at *p* < 0.05. † Adjusted for age, sex, income, region of residence, systolic blood pressure, diastolic blood pressure, fasting blood glucose, total cholesterol, obesity, smoking, alcohol consumption, Charlson Comorbidity Index scores, GERD, and H_2_-receptor antagonist.

**Table 3:** Crude and overlap propensity score weighted odds ratios of H_2_ receptor antagonist (ref: nonuser) for esophageal cancer with subgroup analysis by the numbers of GERD episodes.

Characteristics	N of Esophageal Cancer	N of Control	Odds Ratios for Esophageal Cancer (95% CI)			
	Exposure/Total (%)	Exposure/Total (%)	Crude	*p*	Overlap Weighted Model †	*p*
Total participants (n = 4055)						
Exposure history						
Current	387/811 (47.7)	493/3244 (15.2)	5.51 (4.52–6.72)	<0.001 *	4.34 (3.67–5.14)	<0.001 *
Past	220/811 (27.1)	1318/3244 (40.6)	1.17 (0.96–1.44)	0.128	0.99 (0.84–1.16)	0.871
Duration of H2RA use (days)						
<30	344/811 (42.4)	1133/3244 (34.9)	2.13 (1.76–2.58)	<0.001 *	1.91 (1.64–2.21)	<0.001 *
30–90	144/811 (17.8)	394/3244 (12.1)	2.57 (2.02–3.27)	<0.001 *	1.93 (1.58–2.35)	<0.001 *
≥90	119/811 (14.7)	284/3244 (8.8)	2.94 (2.27–3.82)	<0.001 *	2.09 (1.69–2.59)	<0.001 *
GERD = 0 (n = 3304)						
Exposure history						
Current	230/507 (45.4)	379/2797 (13.6)	5.58 (4.41–7.07)	<0.001 *	4.74 (3.94–5.72)	<0.001 *
Past	129/507 (25.4)	1056/2797 (37.8)	1.12 (0.88–1.44)	0.358	1.01 (0.85–1.20)	0.922
Duration of H2RA use (days)						
<30	223/507 (44.0)	946/2797 (33.8)	2.17 (1.73–2.71)	<0.001 *	2.00 (1.70–2.35)	<0.001 *
30–90	72 /507 (14.2)	293/2797 (10.5)	2.26 (1.66–3.08)	<0.001 *	1.77 (1.41–2.23)	<0.001 *
≥90	64 /507 (12.6)	196/2797 (7.0)	3.00 (2.16–4.18)	<0.001 *	2.51 (1.96–3.22)	<0.001 *
GERD = 1 (n = 281)						
Exposure history						
Current	55/107 (51.4)	45/174 (25.9)	1.32 (0.67–2.61)	0.419	1.49 (0.69–3.24)	0.312
Past	28/107 (26.2)	103/174 (59.2)	0.29 (0.15–0.59)	0.001 *	0.28 (0.13–0.62)	0.002 *
Duration of H2RA use (days)						
<30	50/107 (46.7)	86/174 (49.4)	0.63 (0.33–1.21)	0.167	0.71 (0.34–1.46)	0.350
30–90	20/107 (18.7)	32/174 (18.4)	0.68 (0.31–1.49)	0.332	0.80 (0.33–1.98)	0.635
≥90	13/107 (12.1)	30/174 (17.2)	0.47 (0.20–1.10)	0.083	0.53 (0.21–1.38)	0.196
GERD = 2 (n = 174)						
Exposure history						
Current	41/76 (53.9)	23/98 (23.5)	2.67 (1.15–6.24)	0.023 *	3.22 (0.97–10.66)	0.056
Past	21/76 (27.6)	54/98 (55.1)	0.58 (0.25–1.36)	0.210	0.56 (0.19–1.65)	0.292
Duration of H2RA use (days)						
<30	29/76 (38.2)	39/98 (39.8)	1.12 (0.49–2.56)	0.796	0.79 (0.27–2.32)	0.667
30–90	22/76 (28.9)	30/98 (30.6)	1.10 (0.46–2.63)	0.830	1.61 (0.49–5.25)	0.432
≥90	11/76 (14.5)	8/98 (8.2)	2.06 (0.66–6.41)	0.211	2.15 (0.49–9.38)	0.309
GERD ≥ 3 (n = 296)						
Exposure history						
Current	61/121 (50.4%)	46/175 (26.3)	1.77 (0.86–3.64)	0.121	1.62 (0.77–3.38)	0.202
Past	42/121 (34.7%)	105/175 (60.0)	0.53 (0.26–1.08)	0.082	0.57 (0.28–1.18)	0.131
Duration of H2RA use (days)						
<30	42/121 (34.7%)	62/175 (35.4)	0.90 (0.44–1.87)	0.783	1.32 (0.62–2.79)	0.467
30–90	30/121 (24.8%)	39/175 (22.3)	1.03 (0.47–2.23)	0.949	0.86 (0.38–1.96)	0.726
≥90	31/121 (25.6%)	50/175 (28.6)	0.83 (0.39–1.76)	0.623	0.60 (0.27–1.34)	0.212

Abbreviations: GERD—gastroesophageal reflux disease; N—number; 95% CI—95% confidence interval; H2RA—H_2_ receptor antagonist. * Significance at *p* < 0.05. † Adjusted for age, sex, income, region of residence, systolic blood pressure, diastolic blood pressure, fasting blood glucose, total cholesterol, obesity, smoking, alcohol consumption, Charlson Comorbidity Index scores, GERD, and H_2_-receptor antagonist.

### 2.3. Associations between Previous H2RA Use and Its Duration and Esophageal Cancer

A similar relationship was also found between previous exposure to H2RAs and the development of esophageal cancer. While past use of H2RAs showed no association with the occurrence of esophageal cancer, the current use of H2RAs was related to higher odds for esophageal cancer than in the control nonuser group (aOR 4.34; 95% CI 3.67–5.14; *p* < 0.001). This association remained valid in subgroups with no GERD, at all ages, or in both sexes.

**Figure 2:**
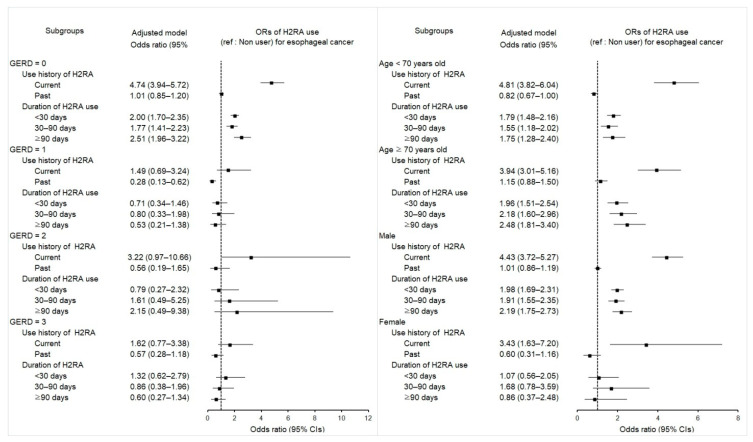
Forest plots for multivariable conditional logistic regression depicting the overlap weighted odds ratios (95% confidence intervals) of previous H2RA exposure history and use duration for incident esophageal cancer according to the number of GERD episodes, age, and sex. The reference is a nonuser. Full results of the crude and adjusted overall weighted models are available in Supplementary Table S3.

Prior use of H2RAs was related to the enhanced likelihood of esophageal cancer, irrelevant of the treatment length in both crude and overlap-weighted models (*p* < 0.001 for all; Table 3). People with prior use of H2RAs demonstrated higher odds for esophageal cancer in those with a period of <30 days (aOR 1.91; 95% CI 1.64–2.21; *p* < 0.001), 30–90 days (aOR 1.93; 95% CI 1.58–2.35; *p* < 0.001), or ≥90 days (aOR 2.09; 95% CI 1.69–2.59; *p* < 0.001). Subgroup analyses advocated the observed negative effect of H2RAs on the incidence of esophageal cancer in patients without GERD episodes, regardless of the treatment duration (Figure 2 and Table S3).

### 2.4. Associations between Mortality in Esophageal Cancer Patients and Use of PPI

**Figure 3:**
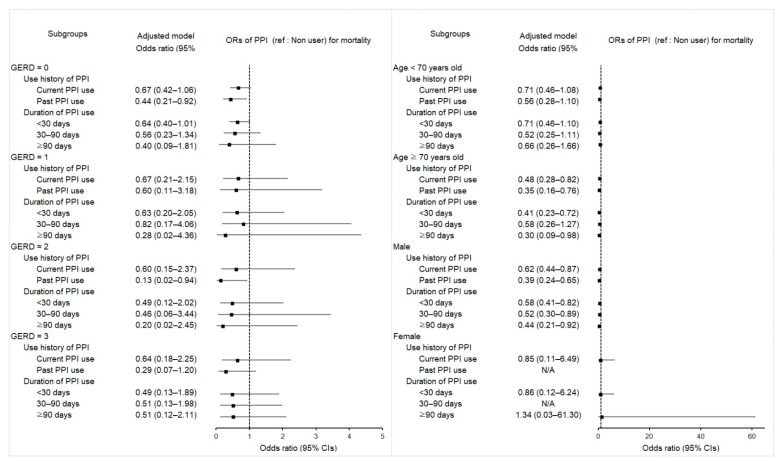
Forest plots for multivariable conditional logistic regression depicting the overlap weighted odds ratios (95% confidence intervals) of previous PPI exposure history and use duration for overall mortality in the patients with incident esophageal cancer according to the number of GERD episodes, age, and sex. The reference is a nonuser. Full results of the crude and adjusted overall weighted models are available in Supplementary Table S4.

After propensity score overlap weighting, both current and past PPI use were related to reduced odds of mortality in patients with esophageal cancer (aOR 0.62; 95% CI 0.45–0.86; *p* = 0.004 and aOR 0.41; 95% CI 0.25–0.67; *p* < 0.001, respectively) (Table 4). Subgroup analyses in patients of the male sex or aged >70 years supported the inverse association between PPI use and mortality, which commonly overlapped in current and past PPI use (Figure 3 and Table S4). 

The probability of mortality was substantially decreased, independent of the total medication days of PPI, in both crude and adjusted models (*p* < 0.005 for all). Patients showed lower ORs for mortality in those with durations of <30 days (aOR 0.58; 95% CI 0.41–0.80; *p* = 0.001), 30–90 days (aOR 0.56; 95% CI 0.34–0.94; *p* = 0.029), or ≥90 days (aOR 0.48; 95% CI 0.24–0.95; *p* = 0.035). Subgroup analyses in patients aged >70 years and men retained the observed effect of PPIs on reducing mortality in esophageal cancer patients.

**Table 4:** Crude and overlap propensity score weighted odds ratios of proton pump inhibitor (ref: nonuser) for mortality in esophageal cancer participants with subgroup analysis by the number of GERD episodes.

Characteristics	Deceased Pts	Survived Pts	Odds Ratios for Mortality (95% Confidence Interval)			
	Exposure/Total (%)	Exposure/Total (%)	Crude	*p*	Overlap Weighted Model †	*p*
Total participants (n = 811)						
Exposure history						
Current	151/470 (32.1)	128/341 (37.5)	0.70 (0.52–0.95)	0.023 *	0.62 (0.45–0.86)	0.004 *
Past	39/470 (8.3)	46/341 (13.5)	0.51 (0.32–0.81)	0.004 *	0.41 (0.25–0.67)	<0.001 *
Duration of PPI use (days)						
<30	123/470 (26.2)	108/341 (31.7)	0.68 (0.49–0.94)	0.019 *	0.58 (0.41–0.80)	0.001 *
30–90	42/470 (8.9)	41/341 (12.0)	0.61 (0.38–0.98)	0.040 *	0.56 (0.34–0.94)	0.029 *
≥90	25/470 (5.3)	25/341 (7.3)	0.60 (0.33–1.07)	0.084	0.48 (0.24–0.95)	0.035 *
GERD = 0 (n = 507)						
Exposure history						
Current	63/315 (20.0)	39/192 (20.3)	0.94 (0.60–1.48)	0.790	0.67 (0.42–1.06)	0.086
Past	15/315 (4.8)	15/192 (7.8)	0.58 (0.28–1.23)	0.155	0.44 (0.21–0.92)	0.030 *
Duration of PPI use (days)						
<30	60/315 (19.0)	39/192 (20.3)	0.90 (0.57–1.41)	0.635	0.64 (0.40–1.01)	0.053
30–90	14/315 (4.4)	12/192 (6.3)	0.68 (0.31–1.51)	0.343	0.56 (0.23–1.34)	0.192
≥90	4/315 (1.3)	3/192 (1.6)	0.78 (0.17–3.52)	0.743	0.40 (0.09–1.81)	0.233
GERD = 1 (n = 107)						
Exposure history						
Current	23/50 (46.0)	33/57 (57.9)	0.58 (0.24–1.38)	0.220	0.67 (0.21–2.15)	0.499
Past	9/50 (18.0)	9/57 (15.8)	0.83 (0.26–2.63)	0.756	0.60 (0.11–3.18)	0.550
Duration of PPI use (days)						
<30	22/50 (44.0)	31/57 (54.4)	0.59 (0.25–1.42)	0.240	0.63 (0.20–2.05)	0.448
30–90	6/50 (12.0)	9/57 (15.8)	0.56 (0.16–1.92)	0.353	0.82 (0.17–4.06)	0.809
≥90	4/50 (8.0)	2/57 (3.5)	1.67 (0.27–10.39)	0.585	0.28 (0.02–4.36)	0.364
GERD = 2 (n = 76)						
Exposure history						
Current	25/42 (59.5)	19/34 (55.9)	0.71 (0.24–2.12)	0.538	0.60 (0.15–2.37)	0.465
Past	4/42 (9.5)	8/34 (23.5)	0.27 (0.06–1.22)	0.089	0.13 (0.02–0.94)	0.043 *
Duration of PPI use (days)						
<30	21/42 (50.0)	17/34 (50.0)	0.67 (0.22–2.04)	0.475	0.49 (0.12–2.02)	0.320
30–90	6/42 (14.3)	6/34 (17.6)	0.54 (0.13–2.31)	0.405	0.46 (0.06–3.44)	0.450
≥90	2/42 (4.8)	4/34 (11.8)	0.27 (0.04–1.86)	0.183	0.20 (0.02–2.45)	0.210
GERD ≥ 3 (n = 121)						
Exposure history						
Current	40/63 (63.5)	37/58 (63.8)	0.63 (0.22–1.77)	0.382	0.64 (0.18–2.25)	0.490
Past	11/63 (17.5)	14/58 (24.1)	0.46 (0.14–1.56)	0.211	0.29 (0.07–1.20)	0.088
Duration of PPI use (days)						
<30	20/63 (31.7)	21/58 (36.2)	0.56 (0.18–1.69)	0.302	0.49 (0.13–1.89)	0.302
30–90	16/63 (25.4)	14/58 (24.1)	0.67 (0.21–2.16)	0.499	0.51 (0.13–1.98)	0.329
≥90	15/63 (23.8)	16/58 (27.6)	0.55 (0.17–1.76)	0.311	0.51 (0.12–2.11)	0.350

Abbreviations: GERD, Gastroesophageal reflux disease; pts, Patients; PPI, Proton pump inhibitor. * Significance at *p* < 0.05. † Adjusted for age, sex, income, region of residence, systolic blood pressure, diastolic blood pressure, fasting blood glucose, total cholesterol, obesity, smoking, alcohol consumption, Charlson Comorbidity Index scores, GERD, and H_2_ receptor antagonist.

### 2.5. Associations between Mortality in Esophageal Cancer Patients and Use of H2RA

The opposite impacts on overall mortality were found between current and past H2RA users (Table 5). The odds of mortality remained significantly higher in patients with a current use history of H2RA (aOR 1.60; 95% CI 1.12–2.28; *p* = 0.010) but conversely lower in those with a past H2RA use history (aOR 0.64; 95% CI 0.43–0.96; *p* = 0.030) than in controls, even after adjusting for potential factors in multivariable logistic regression analysis adjusting all covariates.

**Figure 4:**
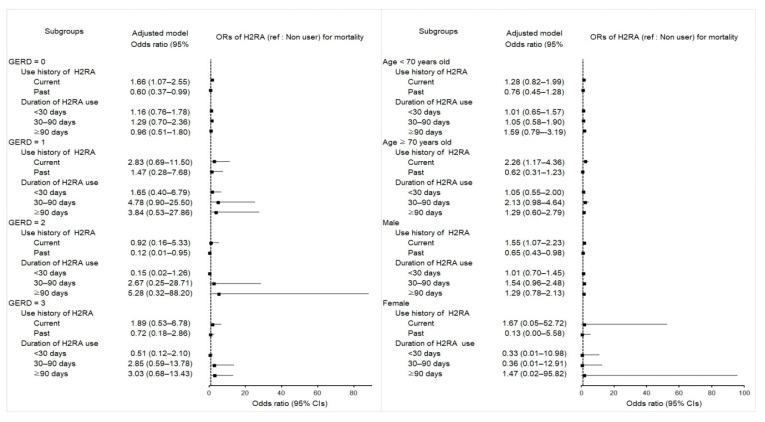
Forest plots for multivariable conditional logistic regression depicting the overlap weighted odds ratios (95% confidence intervals) of previous H2RA e exposure history and use duration for overall mortality in the patients with incident esophageal cancer according to the number of GERD episodes, age, and sex. The reference is a nonuser. Full results of the crude and adjusted overall weighted models are available in Supplementary Table S5.

The profound effect of current H2RA on mortality was observed in elderly male patients without GERD episodes, whereas that of past H2RA was seen in the patients with two episodes of GERD (Figure 4 and Table S5), suggesting that a history of current H2RA exacerbates prognosis in elderly male patients without GERD episodes. However, past H2RA improves prognosis in patients with intermittent episodes of GERD.

**Table 5:** Crude and overlap propensity score weighted odds ratios of H_2_ receptor antagonist (ref: nonuser) for mortality in esophageal cancer participants with subgroup analysis by the number of GERD episodes.

Characteristics	Deceased Pts	Survived Pts	Odds Ratios for Mortality (95% Confidence Interval)			
	Exposure/Total (%)	Exposure/Total (%)	Crude	*p*	Overlap Weighted Model †	*p*
Total participants (n = 811)						
Exposure history						
Current	257/470 (54.7)	130/341 (38.1)	1.66 (1.17–2.34)	0.004 *	1.60 (1.12–2.28)	0.010 *
Past	102/470 (21.7)	118/341 (34.6)	0.72 (0.49–1.06)	0.098	0.64 (0.43–0.96)	0.030 *
Duration of H2RA use (days)						
<30	191/470 (40.6)	153/341 (44.9)	1.05 (0.74–1.48)	0.800	1.02 (0.71–1.45)	0.924
30–90	94/470 (20.0)	50/341 (14.7)	1.58 (1.01–2.45)	0.043 *	1.49 (0.94–2.34)	0.087
≥90	74/470 (15.7)	45/341 (13.2)	1.38 (0.87–2.19)	0.174	1.37 (0.84–2.22)	0.207
GERD = 0 (n = 507)						
Exposure history						
Current	164/315 (52.1)	66/192 (34.4)	1.84 (1.19–2.84)	0.006 *	1.66 (1.07–2.55)	0.022 *
Past	66/315 (21.0)	63/192 (32.8)	0.78 (0.48–1.25)	0.296	0.60 (0.37–0.99)	0.047 *
Duration of H2RA use (days)						
<30	138/315 (43.8)	85/192 (44.3)	1.20 (0.79–1.84)	0.392	1.16 (0.76–1.78)	0.481
30–90	50/315 (15.9)	22/192 (11.5)	1.68 (0.93–3.06)	0.088	1.29 (0.70–2.36)	0.417
≥90	42/315 (13.3)	22/192 (11.5)	1.41 (0.77–2.60)	0.265	0.96 (0.51–1.80)	0.896
GERD = 1 (n = 107)						
Exposure history						
Current	30/50 (60.0)	25/57 (43.9)	2.40 (0.88–6.53)	0.087	2.83 (0.69–11.50)	0.147
Past	12/50 (24.0)	16/57 (28.1)	1.50 (0.48–4.65)	0.483	1.47 (0.28–7.68)	0.645
Duration of H2RA use (days)						
<30	24/50 (48.0)	26/57 (45.6)	1.85 (0.67–5.09)	0.236	1.65 (0.40–6.79)	0.490
30–90	12/50 (24.0)	8/57 (14.0)	3.0(0.87–10.29)	0.081	4.78 (0.90–25.50)	0.067
≥90	6/50 (12.0)	7/57 (12.3)	1.71 (0.43–6.83)	0.445	3.84 (0.53–27.86)	0.183
GERD = 2 (n = 76)						
Exposure history						
Current	27/42 (64.3)	14/34 (41.2)	1.07 (0.30–3.81)	0.915	0.92 (0.16–5.33)	0.924
Past	6/42 (14.3)	15/34 (44.1)	0.22 (0.05–0.94)	0.042 *	0.12 (0.01–0.95)	0.044 *
Duration of H2RA use (days)						
<30	14/42 (33.3)	15/34 (44.1)	0.52 (0.14–1.93)	0.327	0.15 (0.02–1.26)	0.081
30–90	12/42 (28.6)	10/34 (29.4)	0.67 (0.17–2.65)	0.564	2.67 (0.25–28.71)	0.419
≥90	7/42 (16.7)	4/34 (11.8)	0.97 (0.19–5.03)	0.973	5.28 (0.32–88.20)	0.247
GERD ≥ 3 (n = 121)						
Exposure history						
Current	36/63 (57.1)	25/58 (43.1)	1.44 (0.50–4.14)	0.498	1.89 (0.53–6.78)	0.327
Past	18/63 (28.6)	24/58 (41.4)	0.75 (0.25–2.27)	0.611	0.72 (0.18–2.86)	0.645
Duration of H2RA use (days)						
<30	15/63 (23.8)	27/58 (46.6)	0.56 (0.18–1.70)	0.303	0.51 (0.12–2.10)	0.349
30–90	20/63 (31.7)	10/58 (17.2)	2.00 (0.60–6.61)	0.256	2.85 (0.59–13.78)	0.193
≥90	19/63 (30.2)	12/58 (20.7)	1.58 (0.49–5.12)	0.443	3.03 (0.68–13.43)	0.145

Abbreviations: GERD, Gastroesophageal reflux disease; pts, Patients; H2RA, H_2_ receptor antagonist. * Significance at *p* < 0.05. † Adjusted for age, sex, income, region of residence, systolic blood pressure, diastolic blood pressure, fasting blood glucose, total cholesterol, obesity, smoking, alcohol consumption, Charlson Comorbidity Index scores, GERD, and proton pump inhibitor.

## 3. Discussion

In this nationwide population-based cohort study, prior PPI and H2RA use increased the likelihood of esophageal cancers, independent of the period of their medication, which seems to favor a possible connection between PPI/H2RA use and the incident esophageal cancers. Earlier observational studies and meta-analyses appear to emphasize the cancer-protective effect of PPIs that prevents the progression to esophageal cancer [21,22,23], of which the vast majority were implemented in Western countries, including the United States, Europe, and Australia, focusing on the Barrett esophagus, with insufficiently matched control groups [7,21,22,23,24]. On the other hand, current epidemiological studies more likely indicate an increased probability of incident esophageal cancers following the use of PPIs [10,11,12,13,14,15]. One large Swedish population-based study shows that the risk for esophageal cancers is increased, irrespective of the length of PPI administration, through a cohort of nearly 800,000 patients taking PPIs [11]. To date, the Taiwanese case–control study published in 2012 is the only Asian-based study to explore the relationship of PPI with the incidence of esophageal carcinomas [13]; based on 549 patients, PPI users exhibited a 3.83-fold greater risk of esophageal cancer (95% CI 3.01–4.89) than nonusers, whereas H2RA use showed only an increased tendency toward the association without statistical significance (OR 1.21; 95% CI 0.95–1.53) [13]. This study originally focused on the correlation of statin administration with the incidence of esophageal cancers, adjusting for comedications, including PPIs and H2RAs. Because of the discrepancy with Western population-based studies at that time, the observed PPI effect on the risk for esophageal cancers was considered biased by the authors [13]. Furthermore, previous studies on this issue had limitations in corroborating the minor number of patients taking H2RAs [18,19,24,27,31,32]. Interestingly, similar relationships are observed in the present study; current exposure histories of PPI and H2RA exhibited 13.23-fold greater odds (95% CI 10.25–17.06) and 4.34-fold greater odds (95% CI 3.67–5.14) for the incident esophageal cancers than for nonuser groups, respectively, even after full adjustment.

In the present study, the remarkable difference in subanalysis results between current PPI and H2RA use was to be independent of the number of GERD episodes in the former but to be confined to the patients without GERD in the latter, implying that individuals without GERD exposed to current PPI and H2RA use may be likely to develop esophageal cancers. Similarly, one recent study has also shown that PPI users without GERD for <1 year are associated with a high risk of esophageal adenocarcinoma (13.76; 95% CI 12.46–15.15) and squamous cell carcinoma (10.96; 95% CI 9.65–12.40) [11], which may indicate that PPI use without GERD might be at considerably increased probability for esophageal cancers, irrespective of histology. In Korea, most esophageal cancers (97%) are squamous cell carcinoma, and a minor portion is an adenocarcinoma in the histology category [33]. People who have GERD with more frequent symptoms may have a slightly higher risk for adenocarcinoma of the esophagus [4]. Based on our findings on the potential negative effect of PPI and H2RA with esophageal cancer, especially in all adults without GERD, those impacts seem to be more likely related to squamous cell carcinoma.

Meanwhile, there have been few studies examining the pro- or anticarcinogenic effects of H2RAs themselves on esophageal neoplasms concerning GERD episodes. One study predominantly comprising Caucasian patients has reported that H2RA seems to have no effect on the progression of esophageal neoplasm in the patients with Barrett’s esophagus [32]; however, the study did not investigate those without Barrett’s esophagus (that might be presumed as the condition without GERD). Comparing with the abovementioned Taiwanese study, we further estimated the risk difference of esophageal cancer depending on the duration of use and the number of GERD episodes in more detail, based on a homogenous and evenly balanced cohort between groups.

The most plausible theory for the association between PPI use and the development of esophageal cancer may be hypergastrinemia, which has been indicated to be involved in carcinogenesis in many digestive cancers [16]. Since gastrin and its receptors (mediating gastrin effects) are also coexpressed in esophageal cancer and its precursor lesions [16], they are considered to contribute to the development of esophageal cancers. Blocking gastrin suppresses the progress of esophageal carcinoma both in cell line and animal experiments [16]. In fact, PPIs cause a physiologically secondary hypergastrinemia, which can reach extremely high plasma gastrin levels in some PPI-treated patients and is proven to be linked with an enhanced risk of high-grade dysplasia or esophageal cancer in a subset of susceptible patients [16]. The negative impact of current exposure to PPIs relevant to incident esophageal cancers might be presumed that irreversible binding of PPIs to proton pumps of gastric parietal cells [17], where the effect duration on overall gastric pH levels may be consequently long-lasting [9] and even short-term exposure might possibly enable adverse effects. Furthermore, because PPIs are metabolized by hepatic cytochrome p450 [29], the slow metabolizers with their genetic polymorphisms commonly seen in a subset of Asians may achieve higher levels of PPIs even with short-term use and a comparatively low dose of PPIs [16,29]. Since H2RAs decrease acid suppression by blocking the effects of histamine only, they may be less effective than PPIs; less likely to induce hypergastrinemia, and less likely to be associated with an increased risk of esophageal cancer from a theoretical aspect. This might partially explain the much higher ORs for the incident esophageal cancers when using PPIs than H2RA in the present study.

The effect of PPIs or H2RAs on the subsequent mortality of patients with cancer is largely unknown. Our study indicated a reduced probability of mortality of esophageal cancers in current and past PPI users by 38% (95% CI 0.45–0.86) and 59% (95% CI 0.25–0.67), respectively. However, these mortality benefits of prior PPI users contrast with the findings of an increase in the incidence of esophageal cancer. Explainable clues may be found in experimental studies of the double-sided properties of PPIs on cancer cells [28,34,35]. PPIs appear to have bidirectional effects depending on primary vs. metastatic esophageal tumor cells [28]. Esomeprazole induces autophagy in primary esophageal cancer cells [28], which allows proliferative mechanisms in cancer cells. Autophagy is an adaptive mechanism that plays a role in malignant cell survival in unwilling circumstances such as hypoxia, nutrient starvation, or cytotoxic drugs [34,35]. In metastatic cells, esomeprazole blocks basal autophagy [28,34], which may inhibit metastatic tumor cell proliferation. Similar findings to our study may be observed in a limited cohort study [20]. Cancer mortality risk associated with PPI use is lowest for esophageal cancer (0.91; 95% CI 0.81–1.04) but highest for ovarian cancer (1.35; 95% CI 1.20–1.52), albeit with postdiagnostic PPI use for esophageal cancer [20]. Otherwise, there is no epidemiologic study specifically investigating the connections between prior PPI/H2RA use and the subsequent mortality in patients suffering from esophageal cancer. Because of insufficient studies, we could not explain paradoxical relationships of current and past exposures H2RAs on mortality of esophageal cancer; current exposure exhibited 1.60-fold greater odds for death (95% CI 1.12–2.28) in subgroups of elderly male patients (>70 years) without GERD. Conversely, its past exposure showed a protective effect related to the 36% reduced probability (95% CI 0.43–0.96) in the subgroup of patients suffering intermittent episodes of GERD. The disparity in effects of H2RA at different exposure histories might be mediated by certain risk factors, including the presence of GERD and old age. Theoretically, H_2_ receptors are mainly distributed in the myocardium of the right atrium and ventricle in animal studies [36]. Functional or structural alterations of H_2_ receptors in cardiomyocytes may lead to cardiac arrhythmias [37]. Thus, the negative impact of H2RA might more likely happen in elderly patients, which might eventually indirectly increase the patients’ death ratio. The association between H2RA and mortality needs further research.

The strength of this study is based first on a representative nationwide cohort database with balanced patients and control members, which draw our findings more generalizable. Because the Korean National Health Insurance Service-Health Screening Cohort (KNHIS-HSC) data included every hospital and clinic in the whole country without exceptions, no medical history was missed during follow-up. Second, we gave a careful consideration to potential confounders. The fully balanced adjustments of socioeconomic position and possible risk factors and comorbidities associated with esophageal carcinoma or PPI or H2RA users (e.g., total cholesterol level, alcohol consumption, smoking status, blood pressure, obesity status, and fasting blood glucose level) may be a further advantage. Third, given the scarce qualified studies enrolling patients taking H2RA in this issue, our study is highlighted by the largest cohort enrolling H2RA users.

This study had some limitations that should be addressed. First, no information referring to *H. pylori*, stage, histology and differentiation, family history, and genetic data of esophageal cancer was incorporated in the health insurance dataset, so the possibility of missing data was not taken into consideration. Second, patient adherence to medication could not be verified using the KNHIS-HSC data. Third, as this study enrolled patients according to diagnosis codes and comprised only Korean subjects, unmeasured confounding effects could not be absolutely eliminated. Fourth, the Korean-based data might not encompass other Asian populations because of a wide spectrum of Asia geographic variation in their incidence and relative prevalence.

## 4. Materials and Methods

### 4.1. Study Population and Participant Selection

The Institutional Review Board of Hallym University (23 October 2019) permitted this study and waived the prerequisite for written informed consent. The study was carried out in proportion to the regulations of the ethics committee of Hallym University.

This study is a retrospective, nested case–control one, using the Korean National Health Insurance Service-Health Screening Cohort (KNHIS-HSC) data, which offers population-based data on the Koreans for research purposes. The KNHIS is a mandatory national health insurance policy in Korea, covering more than 98% of Korean citizens since 1999. The medical data files available and all individuals’ information were de-identified and anonymized [38]. The diagnostic codes used in the KNHIS-HSC database follow the International Classification of Diseases, 10th Revision, Clinical Modification (ICD-10-CM). A detailed depiction of the KNHIS-HSC data has been explained previously [39].

**Figure 5:**
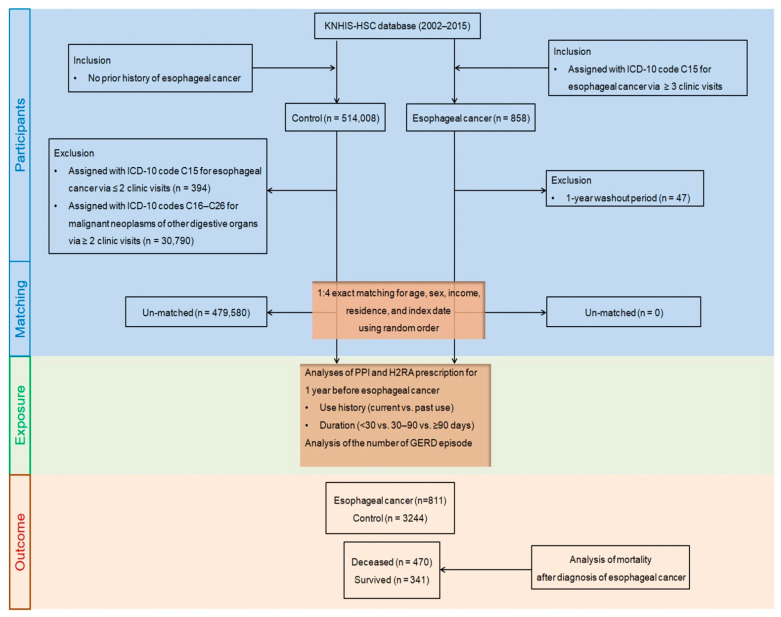
A schematic illustration of the participant selection process that was used in the present study. From the KNHIS-HSC database, a total of 811 esophageal cancer participants were matched with 3244 control participants for age, sex, income, region of residence, and index date.

A total of 858 newly diagnosed patients with esophageal cancer between 2002 and 2015 were initially extracted from the KNHIS-HSC database, which harbors 514,866 participants aged more than 40 years with 615,488,428 medical claim codes (Figure 5). To lessen any incidences of false positives, esophageal carcinoma was retrieved using ICD-10 code C15 (malignant neoplasm of the esophagus) with more than three clinic visits for diagnostic assessment histories. The index date of every esophageal cancer patient was established as the day when the ICD-10 code for esophageal cancer (C15) was electronically arranged for participants in health insurance claims datasets. We excluded the patients diagnosed with esophageal cancer in 2002 (1-year washout period, n = 47), as we possibly might include pre-existing esophageal cancer before the index date in the analysis.

The control cohort who had never been diagnosed with esophageal cancer was retrieved from the database from 2002 to 2015 (n = 514,008). The control members were eliminated if they had been diagnosed with esophageal cancer (C15) with ≤two clinic visits (n = 394) or diagnosed with other malignant neoplasms of digestive organs (ICD-10 codes: C16–C26) with >two clinic visits with an assigned code (n = 30,790), such as malignant neoplasms of the stomach (C16), small intestine (C17), colon (C18), rectosigmoid junction (C19), rectum (C20), anus and anal canal (C21), liver and intrahepatic bile ducts (C22), gallbladder (C23), other and unspecified parts of the biliary tract (C24), pancreas (C25), and other and ill-defined digestive organs (C26).

To optimize the balance of baseline characteristics between esophageal cancer and control members, propensity score matching was applied on the basis of age, sex, income, and residence, using random clustered sampling to reduce possible selection bias. The index date of the control participants followed the index date of their matched esophageal cancer patients. Therefore, every matched esophageal cancer patient with a control member had an identical index date. Through the matching steps, 479,580 control members were eventually unmatched and eliminated. Therefore, 811 patients with esophageal cancer were matched with 3244 control members at a ratio of 1:4.

### 4.2. Exposures (PPIs and H2RAs)

We then retrospectively identified the PPI and H2RA prescription period for one year before the diagnosis of esophageal cancer in the cohort groups. Only participants with the first use of PPIs or H2RAs within one year prior to the index date were qualified for the study. KNHIS-HSC data cover information on prescription drugs, including drug code, drug name, date of prescription, daily dose, and period. We gathered the prescription data of each participant for seven supplied kinds of PPI (lansoprazole, omeprazole, rabeprazole, pantoprazole, ilaprazole, esomeprazole, and dexlansoprazole) and six kinds of H2RA (ranitidine, cimetidine, famotidine, nizatidine, roxatidine, and lafutidine). The history of PPI or H2RA use was based on the prescription and assorted as current exposure (prescribed at least once within the last 30 days) and past exposure (prescribed at least once within the last 31–365 days) to examine the effect of the temporality of PPI or H2RA use on cancer risk. To estimate the cumulative impact of PPI or H2RA exposure, the cumulative duration of total drug use was measured as the overall prescription date in the year preceding the index date and categorized as <30 days, 30 to 90 days, and ≥90 days.

### 4.3. Outcome (Esophageal Cancer)

Esophageal cancer was designated on the basis of the ICD-10 code (C15) that was assigned to more than three clinic visits. The primary outcome was the occurrence of esophageal cancer with the former use history (current vs. past use) and duration (<30 days vs. 30–90 days vs. ≥90 days) of PPIs or H2RAs, depending on the number of GERD episodes. The secondary outcome was the odds of death (overall or all-cause mortality) from these patients based on PPI or H2RA use and the number of GERD episodes.

The patients were divided according to treatment modality as participants who did not receive any medical treatment, those who only underwent surgery (claim code: Q2390–Q2392, Q2401–Q2403), or underwent surgery with adjuvant therapy, including chemotherapy or radiotherapy.

### 4.4. Covariates

The people were categorized into 10 age groups depending on 5-year intervals and five income groups—class 1 (lowest income) to class 5 (highest income). The area of residence was asserted on the basis of urban and rural areas, as previously described [40]. Obesity status using body mass index (kg/m^2^), smoking, and alcohol consumption were classified in an assessment similar to our previous study [41]. The total cholesterol (mg/dL), fasting blood glucose (mg/dL), diastolic blood pressure (mmHg), and systolic blood pressure (mmHg) were collected. The Charlson Comorbidity Index (CCI) was aggregated as a total score from 0 (no comorbidities) to 29 (multiple comorbidities) to quantify the severity and number of diseases using 17 comorbidities [42]. The CCI score was computed without including esophageal carcinoma. The number of GERD episodes (determined as the ICD-10 code [K21] with ≥2 clinic visits and prescription of PPI for ≥2 weeks) for the year before the index date was additionally evaluated. We adjusted the potential confounding factors of age, sex, income, residence, obesity, smoking, alcohol, systolic or diastolic blood pressure, fasting blood glucose, total cholesterol, GERD, and CCI scores using overlap-weighted models by multivariable conditional logistic regression (when we analyzed PPI, H2RA was adjusted as covariates and vice versa).

### 4.5. Statistical Analyses

Categorical data are summarized as percentages. Continuous data are presented as the mean and standard deviation. We implemented propensity score overlap weighting to optimize covariate balance and effective sample size. The propensity score was computed using multivariable logistic regression analysis with all covariates. During the propensity score matching, a greedy, nearest-neighbor matching algorithm was used to form pairs of esophageal carcinoma and control members. Propensity scores where esophageal carcinoma patients and control members were weighted by the probability of a 1-propensity score and the probability of a propensity score, respectively, were subject to overlap weighting estimated between 0 and 1, reflecting the achievement of exact balance and optimized precision [43,44]. To diminish certain bias between the groups, we inspected the balance of the matched data with regard to absolute standardized differences of covariates before and after matching. An absolute standardized difference of <0.20 indicates a good balance for a particular covariate [45]. Propensity score overlap weighted multivariable logistic regressions for crude (unadjusted) and overlap weighted (adjusted for all covariates) models were used to estimate overlap weighted ORs and 95% CIs for incident esophageal carcinoma and their death in terms of the history of PPI or H2RA use and their duration by adjusting for potential confounders. Subgroup analyses were implemented following the number of GERD episodes, age, and sex.

Two-tailed analyses were used, with statistically significant *p*-values less than 0.05. SAS version 9.4 (SAS Institute Inc., Cary, NC, USA) was used for all statistical analyses.

## 5. Conclusions

This nationwide population-based cohort study may carefully speculate on the possible link of PPI/H2RA with incident esophageal cancer in the Korean population.

## Figures and Tables

**Table 1:** Baseline characteristics of esophageal cancer participants after propensity score overlap weighting adjustment.

Characteristics	After PS Overlap Weighting Adjustment			After PS Overlap Weighting Adjustment in Esophageal Cancer Pts		
	Esophageal Cancer	Control	SMD	Deceased pts	Survived pts	SMD
Total participants (n, %)	811 (100)	3244 (100)		341 (100)	470 (100)	
Age, %			0.00			0.00
40–44	0.21	0.21		0.10	0.10	
45–49	1.54	1.54		1.47	1.47	
50–54	6.25	6.25		6.51	6.51	
55–59	10.90	10.90		11.59	11.59	
60–64	15.43	15.43		15.94	15.94	
65–69	22.77	22.77		22.12	22.12	
70–74	20.56	20.56		20.90	20.90	
75–79	15.14	15.14		14.52	14.52	
80–84	5.59	5.59		5.21	5.21	
85+	1.60	1.60		1.64	1.64	
Sex, %			0.00			0.00
Male	92.98	92.98		93.46	93.46	
Female	7.02	7.02		6.54	6.54	
Income, %			0.00			0.00
1 (lowest)	16.94	16.94		16.71	16.71	
2	14.10	14.10		12.56	12.56	
3	16.13	16.13		17.27	17.27	
4	21.92	21.92		23.55	23.55	
5 (highest)	30.92	30.92		29.91	29.91	
Region of residence, %			0.00			0.00
Urban	38.42	38.42		39.55	39.55	
Rural	61.58	61.58		60.45	60.45	
Obesity †, %			0.00			0.00
Underweight	5.82	5.82		5.95	5.95	
Normal	46.86	46.86		51.63	51.63	
Overweight	25.34	25.34		23.80	23.80	
Obese I	21.00	21.00		18.02	18.02	
Obese II	0.98	0.98		0.60	0.60	
Smoking status, %			0.00			0.00
Nonsmoker	42.92	42.92		38.91	38.91	
Past smoker	22.50	22.50		21.92	21.92	
Current smoker	34.58	34.58		39.17	39.17	
Alcohol consumption, %			0.00			0.00
<1 time a week	46.71	46.71		42.65	42.65	
≥1 time a week	53.29	53.29		57.35	57.35	0.00
SBP (Mean, SD)	129.53 (14.48)	129.53 (6.73)	0.00	129.41 (10.10)	129.41 (12.64)	0.00
DBP (Mean, SD)	78.79 (8.57)	78.79 (4.18)	0.00	78.72 (5.75)	78.72 (7.78)	0.00
Fasting blood glucose (Mean, SD)	104.48 (22.57)	104.48 (12.57)	0.00	104.23 (15.74)	104.23 (19.57)	0.00
Total cholesterol (Mean, SD)	188.18 (30.98)	188.18 (14.88)	0.00	185.96 (21.90)	185.96 (26.55)	0.00
CCI score (Mean, SD)	2.38 (1.93)	2.38 (1.05)	0.00	3.14 (1.51)	3.14 (1.79)	0.00
GERD episodes for 1 year before index date (Mean, SD)	0.93 (1.48)	0.93 (1.12)	0.00	1.27 (1.38)	1.27 (1.50)	0.00
Treatment type, %						0.00
No records of treatment	-	-		34.65	34.65	
Surgery only	-	-		16.04	16.04	
Surgery + RT or CT	-	-		49.31	49.31	

Abbreviations: PS—propensity score; SMD—standardized mean difference; pts—patients; CCI—Charlson Comorbidity Index; SBP—systolic blood pressure; DBP—diastolic blood pressure; SD—standard deviation; GERD—gastroesophageal reflux disease; CT—chemotherapy; RT—radiotherapy. † Obesity (BMI—body mass index, kg/m^2^) was categorized as <18.5 (underweight), ≥18.5 to <23 (normal), ≥23 to <25 (overweight), ≥25 to <30 (obese I), and ≥30 (obese II).

## Data Availability

All data are available from the database of the National Health Insurance Sharing Service (NHISS) https://nhiss.nhis.or.kr/ (accessed on 1 January 2020) NHISS allows access to all of this data for any researcher who promises to follow the research ethics at some cost. Those seeking access to this articles’ data can download it from the website after promising to follow the research ethics.

## Supplementary Materials

The following are available online at https://www.mdpi.com/article/10.3390/ph15050517/s1, Table S1: general characteristics of participants before propensity score overlap weighting adjustment, Table S2: subgroup analyses of crude and overlap propensity score weighted odds ratios of proton pump inhibitor (ref: nonuser) for esophageal cancer, Table S3: subgroup analyses of crude and overlap propensity score weighted odds ratios of H2RA (ref: nonuser) for esophageal cancer, Table S4: subgroup analyses of crude and overlap propensity score weighted odds ratios of proton pump inhibitor (ref: nonuser) for mortality in esophageal cancer participants, Table S5: subgroup analyses of crude and overlap propensity score weighted odds ratios of H2RA (ref: nonuser) for mortality in esophageal cancer participants.
