# Supplementary material for: Possible Association between the Use of Proton Pump Inhibitors and H2 Receptor Antagonists, and Esophageal Cancer: A Nested Case–Control Study Using a Korean National Health Screening Cohort
Source: Pharmaceuticals (Basel). 2022 Apr 22;15(5):517. doi: 10.3390/ph15050517 (PMC9146181; doi:10.3390/ph15050517)
Supplement: Supplementary file 1 [file pharmaceuticals-15-00517-s001.zip › pharmaceuticals-1659425-supplementary.pdf]

Table S1. General Characteristics of Participants before propensity score overlap weighting adjustment

Characteristics	Before PS Overlap weighting adjustment			Before PS Overlap weighting adjustment in Esophageal cancer participants		
	Esophageal cancer	Control	SMD	Deceased pts	Survived pts	SMD
Total participants (n, %)	811 (100%)	3,244 (100%)		341 (100%)	470 (100%)	
Age (%)			0.00			0.34
40-44	0.25	0.25		0.21	0.29	
45-49	1.85	1.85		1.49	2.35	
50-54	7.03	7.03		5.96	8.50	
55-59	11.71	11.71		8.72	15.84	
60-64	16.52	16.52		14.89	18.77	
65-69	22.44	22.44		23.19	21.41	
70-74	19.73	19.73		21.49	17.30	
75-79	14.06	14.06		16.38	10.85	
80-84	5.06	5.06		6.17	3.52	
85+	1.36	1.36		1.49	1.17	
Sex (%)			0.00			0.34
Male	93.22	93.22		94.89	90.91	
Female	6.78	6.78		5.11	9.09	
Income (%)			0.00			0.22
1 (lowest)	16.52	16.52		18.72	13.49	
2	14.06	14.06		15.74	11.73	
3	16.77	16.77		17.23	16.13	
4	22.19	22.19		20.21	24.93	
5 (highest)	30.46	30.46		28.09	33.72	
Region of residence (%)			0.00			0.09
Urban	37.85	37.85		35.96	40.47	
Rural	62.15	62.15		64.04	59.53	
Obesity † (%)			0.42			0.42
Underweight	7.77	3.14		10.85	3.52	
Normal	49.69	35.76		53.83	43.99	
Overweight	23.67	27.9		20.64	27.86	
Obese I	18.13	31.13		13.83	24.05	
Obese II	0.74	2.07		0.85	0.59	
Smoking status (%)			0.36			0.24
Nonsmoker	38.22	54.59		37.02	39.88	
Past smoker	21.95	20.59		18.72	26.39	
Current smoker	39.83	24.82		44.26	33.72	

Alcohol consumption (%)			0.23			0.13
<1 time a week	43.03	54.59		45.74	39.30	
≥1 time a week	56.97	45.41		54.26	60.7	
SBP (Mean, SD)	129.46 (18.45)	130.15 (17.05)	0.04	130.49 (18.32)	128.05 (18.56)	0.13
DBP (Mean, SD)	78.77 (10.92)	79.71 (10.74)	0.09	79.01 (10.49)	78.45 (11.49)	0.05
Fasting blood glucose (Mean, SD)	103.92 (28.63)	104.31 (32.15)	0.01	103.40 (27.77)	104.65 (29.79)	0.04
Total cholesterol (Mean, SD)	186.09 (39.80)	193.70 (37.79)	0.20	184.38 (40.03)	188.45 (39.43)	0.10
CCI score (Mean, SD)	3.39 (2.90)	1.12 (1.83)	0.94	4.19 (2.96)	2.29 (2.43)	0.70
The number of GERD for 1 year before index date (Mean, SD)	1.21 (2.58)	0.44 (1.66)	0.36	1.08 (2.31)	1.40 (2.90)	0.12
Treatment type (%)						0.60
No records of treatment	-	-		30.64	39.59	
Surgery only	-	-		8.51	25.51	
Surgery+RT or CT	-	-		60.85	34.90	
The days of H2RA for 1 year before index date (Mean, SD)	45.65 (82.80)	28.24 (65.46)	0.23	47.15 (80.30)	43.58 (86.21)	0.04
The days of PPI use for 1 year before index date (Mean, SD)	19.48 (49.34)	8.23 (34.49)	0.26	15.49 (39.70)	24.98 (59.78)	0.19

Abbreviations: PS, Propensity score; SMD, Standardized mean difference; pts, Patients; CCI, Charlson Comorbidity Index; SBP, Systolic blood pressure; DBP, Diastolic blood pressure; SD, Standard deviation; GERD, Gastroesophageal reflux disease; CT, Chemotherapy; RT, Radiotherapy; H2RA, H_2 receptor antagonist; PPI, proton pump inhibitor.

†Obesity (BMI, body mass index, kg/m^2) was categorized as < 18.5 (underweight), ≥ 18.5 to < 23 (normal), ≥ 23 to < 25 (overweight), ≥ 25 to < 30 (obese I), and ≥ 30 (obese II).

Table S2. Subgroup analyses of crude and overlap propensity score weighted odd ratios of proton pump inhibitor (ref: non user) for Esophageal cancer

Characteristics	N of Esophageal cancer	N of Control	Odd ratios for Esophageal cancer (95% confidence interval)			
	(exposure / total, %)	(exposure / total, %)	Crude	P-value	Overlap weighted model†	P-value
Age < 70 years old (n = 2,425)						
User of PPI						
Current PPI	164 / 485 (33.8%)	50 / 1940 (2.6%)	20.32 (14.44–28.60)	<0.001*	13.39 (9.43–19.00)	<0.001*
Past PPI	49 / 485 (10.1%)	204 / 1940 (10.5%)	1.49 (1.06–2.09)	0.021*	1.06 (0.80–1.39)	0.699
Duration of PPI use						
< 30 days	145 / 485 (29.9%)	149 / 1940 (7.7%)	6.03 (4.64–7.84)	<0.001*	4.26 (3.34–5.42)	<0.001*
30 to 90 days	42 / 485 (8.7%)	68 / 1940 (3.5%)	3.83 (2.55–5.74)	<0.001*	2.52 (1.78–3.59)	<0.001*
≥ 90 days	26 / 485 (5.4%)	37 / 1940 (1.9%)	4.36 (2.60–7.31)	<0.001*	1.75 (1.06–2.88)	0.028*
Age ≥ 70 years old (n = 1,630)						
User of PPI						
Current PPI	115 / 326 (35.3%)	57 / 1304 (4.4%)	12.39 (8.69–17.68)	<0.001*	13.41 (9.05–19.89)	<0.001*
Past PPI	36 / 326 (11.0%)	172 / 1304 (13.2%)	1.29 (0.87–1.90)	0.210	1.19 (0.86–1.64)	0.287
Duration of PPI use						
< 30 days	86 / 326 (26.4%)	104 / 1304 (8.0%)	5.08 (3.66–7.05)	<0.001*	4.80 (3.55–6.49)	<0.001*
30 to 90 days	41 / 326 (12.6%)	73 / 1304 (5.6%)	3.45 (2.28–5.22)	<0.001*	2.94 (2.01–4.31)	<0.001*
≥ 90 days	24 / 326 (7.4%)	52 / 1304 (4.0%)	2.84 (1.70–4.72)	<0.001*	1.97 (1.20–3.25)	0.008*
Male (n = 3,780)						
User of PPI						
Current PPI	262 / 756 (34.7%)	99 / 3024 (3.3%)	16.48 (12.79–21.23)	<0.001*	13.57 (10.43–17.65)	<0.001*
Past PPI	81 / 756 (10.7%)	353 / 3024 (11.7%)	1.43 (1.10–1.86)	0.008*	1.22 (0.99–1.51)	0.061
Duration of PPI use						
< 30 days	219 / 756 (29.0%)	230 / 3024 (7.6%)	5.93 (4.80–7.33)	<0.001*	4.94 (4.06–6.00)	<0.001*
30 to 90 days	80 / 756 (10.6%)	136 / 3024 (4.5%)	3.66 (2.73–4.92)	<0.001*	2.81 (2.16–3.64)	<0.001*
≥ 90 days	44 / 756 (5.8%)	86 / 3024 (2.8%)	3.19 (2.18–4.65)	<0.001*	1.65 (1.16–2.35)	0.005*
Female (n = 275)						
User of PPI						
Current PPI	17 / 55 (30.9%)	8 / 220 (3.6%)	11.81 (4.73–29.53)	<0.001*	14.21 (4.58–44.07)	<0.001*
Past PPI	4 / 55 (7.3%)	23 / 220 (10.5%)	0.97 (0.31–2.97)	0.953	0.31 (0.11–0.90)	0.032*
Duration of PPI use						
< 30 days	12 / 55 (21.8%)	23 / 220 (10.5%)	2.90 (1.32–6.38)	0.008*	1.73 (0.83–3.59)	0.144
30 to 90 days	3 / 55 (5.5%)	5 / 220 (2.3%)	3.34 (0.76–14.61)	0.110	4.17 (1.17–14.88)	0.028*
≥ 90 days	6 / 55 (10.9%)	3 / 220 (1.4%)	11.12 (2.65–46.61)	0.001*	9.57 (1.59–57.50)	0.014*

Abbreviations: GERD, Gastroesophageal reflux disease; N, number; 95% CI, 95% confidence interval; PPI, proton pump inhibitor.

* Significance at $P < 0.05$ †Adjusted for age, sex, income, region of residence, systolic blood pressure, diastolic blood pressure, fasting blood glucose, total cholesterol, obesity, smoking, alcohol consumption, Charlson Comorbidity Index scores, GERD, and H_2 receptor antagonist

Table S3. Subgroup analyses of crude and overlap propensity score weighted odd ratios of H2RA (ref: non user) for Esophageal cancer

Characteristics	N of Esophageal cancer	N of Control	Odd ratios for Esophageal cancer (95% confidence interval)			
	(exposure / total, %)	(exposure / total, %)	Crude	P-value	Overlap weighted model †	P-value
Age < 70 years old (n = 2425)						
Exposure history						
Current H2RA	223 / 485 (46.0%)	218 / 1940 (11.2%)	6.98 (5.39–9.03)	<0.001*	4.81 (3.82–6.04)	<0.001*
Past H2RA	122 / 485 (25.2%)	767 / 1940 (39.5%)	1.09 (0.84–1.41)	0.540	0.82 (0.67–1.00)	0.050
Duration of H2RA use						
< 30 days	219 / 485 (45.2%)	685 / 1940 (35.3%)	2.18 (1.73–2.75)	<0.001*	1.79 (1.48–2.16)	<0.001*
30 to 90 days	74 / 485 (15.3%)	184 / 1940 (9.5%)	2.74 (1.99–3.79)	<0.001*	1.55 (1.18–2.02)	0.002*
≥ 90 days	52 / 485 (10.7%)	116 / 1940 (6.0%)	3.06 (2.11–4.44)	<0.001*	1.75 (1.28–2.40)	0.001*
Age ≥ 70 years old (n = 1630)						
Exposure history						
Current H2RA	164 / 326 (50.3%)	275 / 1304 (21.1%)	4.45 (3.22–6.16)	<0.001*	3.94 (3.01–5.16)	<0.001*
Past H2RA	98 / 326 (30.1%)	551 / 1304 (42.3%)	1.33 (0.95–1.86)	0.100	1.15 (0.88–1.50)	0.303
Duration of H2RA use						
< 30 days	125 / 326 (38.3%)	448 / 1304 (34.4%)	2.08 (1.50–2.89)	<0.001*	1.96 (1.51–2.54)	<0.001*
30 to 90 days	70 / 326 (21.5%)	210 / 1304 (16.1%)	2.49 (1.71–3.63)	<0.001*	2.18 (1.60–2.96)	<0.001*
≥ 90 days	67 / 326 (20.6%)	168 / 1304 (12.9%)	2.98 (2.03–4.38)	<0.001*	2.48 (1.81–3.40)	<0.001*
Male (n = 3780)						
Exposure history						
Current H2RA	363 / 756 (48.0%)	459 / 3024 (15.2%)	5.59 (4.56–6.86)	<0.001*	4.43 (3.72–5.27)	<0.001*
Past H2RA	201 / 756 (26.6%)	1207 / 3024(39.9%)	1.18 (0.95–1.46)	0.131	1.01 (0.86–1.19)	0.929
Duration of H2RA use						
< 30 days	326 / 756 (43.1%)	1047 / 3024(34.6%)	2.20 (1.81–2.68)	<0.001*	1.98 (1.69–2.31)	<0.001*
30 to 90 days	128 / 756 (16.9%)	360 / 3024 (11.9%)	2.51 (1.95–3.24)	<0.001*	1.91 (1.55–2.35)	<0.001*
≥ 90 days	110 / 756 (14.6%)	259 / 3024 (8.6%)	3.00 (2.29–3.93)	<0.001*	2.19 (1.75–2.73)	<0.001*
Female (n = 275)						
Exposure history						
Current H2RA	24 / 55 (43.6%)	34 / 220 (15.5%)	4.41 (1.98–9.85)	<0.001*	3.43 (1.63–7.20)	0.001*
Past H2RA	19 / 55 (34.5%)	111 / 220 (50.5%)	1.07 (0.49–2.33)	0.865	0.60 (0.31–1.16)	0.129
Duration of H2RA use						
< 30 days	18 / 55 (32.7%)	86 / 220 (39.1%)	1.31 (0.59–2.89)	0.507	1.07 (0.56–2.05)	0.828
30 to 90 days	16 / 55 (29.1%)	34 / 220 (15.5%)	2.94 (1.26–6.89)	0.013*	1.68 (0.78–3.59)	0.184
≥ 90 days	9 / 55 (16.4%)	25 / 220 (11.4%)	2.25 (0.85–5.97)	0.103	0.96 (0.37–2.48)	0.934

Abbreviations: GERD, Gastroesophageal reflux disease; N, number; 95% CI, 95% confidence interval; H_2 receptor antagonist, H2RA.

* Significance at $P < 0.05$ †Adjusted for age, sex, income, region of residence, systolic blood pressure, diastolic blood pressure, fasting blood glucose, total cholesterol, obesity, smoking, alcohol consumption, Charlson Comorbidity Index scores, GERD, and H_2 receptor antagonist.

Table S4. Subgroup analyses of crude and overlap propensity score weighted odd ratios of proton pump inhibitor (ref: non user) for mortality in Esophageal cancer participants

Characteristics	Dead participants	Survived participants	Odd ratios for mortality (95% confidence interval)			
	(exposure / total, %)	(exposure/total, %)	Crude	P-value	Overlap weighted model †	P-value
Age < 70 years old (n = 485)						
Exposure history						
Current PPI	84 / 256 (32.8%)	80 / 229 (34.9%)	0.84 (0.57–1.24)	0.384	0.71 (0.46–1.08)	0.112
Past PPI	21 / 256 (8.2%)	28 / 229 (12.2%)	0.60 (0.33–1.11)	0.104	0.56 (0.28–1.10)	0.094
Duration of PPI use						
< 30 days	76 / 256 (29.7%)	69 / 229 (30.1%)	0.88 (0.59–1.32)	0.545	0.71 (0.46–1.10)	0.125
30 to 90 days	17 / 256 (6.6%)	25 / 229 (10.9%)	0.54 (0.28–1.06)	0.072	0.52 (0.25–1.11)	0.092
≥ 90 days	12 / 256 (4.7%)	14 / 229 (6.1%)	0.69 (0.31–1.54)	0.362	0.66 (0.26–1.66)	0.372
Age ≥ 70 years old (n = 326)						
Exposure history						
Current PPI	67 / 214 (31.3%)	48 / 112 (42.9%)	0.50 (0.30–0.82)	0.006*	0.48 (0.28–0.82)	0.008*
Past PPI	18 / 214 (8.4%)	18 / 112 (16.1%)	0.36 (0.17–0.74)	0.006*	0.35 (0.16–0.76)	0.008*
Duration of PPI use						
< 30 days	47 / 214 (22.0%)	39 / 112 (34.8%)	0.43 (0.25–0.74)	0.002*	0.41 (0.23–0.72)	0.002*
30 to 90 days	25 / 214 (11.7%)	16 / 112 (14.3%)	0.56 (0.27–1.14)	0.108	0.58 (0.26–1.27)	0.173
≥ 90 days	13 / 214 (6.1%)	11 / 112 (9.8%)	0.42 (0.18–1.01)	0.052	0.30 (0.09–0.98)	0.046*
Male (n = 756)						
Exposure history						
Current PPI	144 / 446 (32.3%)	118 / 310 (38.1%)	0.67 (0.49–0.92)	0.015*	0.62 (0.44–0.87)	0.006*
Past PPI	36 / 446 (8.1%)	45 / 310 (14.5%)	0.44 (0.27–0.72)	0.001*	0.39 (0.24–0.65)	<0.001*
Duration of PPI use						
< 30 days	118 / 446 (26.5%)	101 / 310 (32.6%)	0.65 (0.46–0.90)	0.010*	0.58 (0.41–0.82)	0.002*
30 to 90 days	39 / 446 (8.7%)	41 / 310 (13.2%)	0.53 (0.32–0.85)	0.009*	0.52 (0.30–0.89)	0.017*
≥ 90 days	23 / 446 (5.2%)	21 / 310 (6.8%)	0.61 (0.32–1.13)	0.115	0.44 (0.21–0.92)	0.029*
Female (n = 55)						
Exposure history						
Current PPI	7 / 24 (29.2%)	10 / 31 (32.3%)	1.00 (0.31–3.26)	1.000	0.85 (0.11–6.49)	0.873
Past PPI	3 / 24 (12.5%)	1 / 31 (3.2%)	4.29 (0.40–45.57)	0.228	N/A	
Duration of PPI use						
< 30 days	5 / 24 (20.8%)	7 / 31 (22.6%)	1.02 (0.27–3.88)	0.976	0.86 (0.12–6.24)	0.882
30 to 90 days	3 / 24 (12.5%)	0 / 31 (0.0%)	N/A		N/A	
≥ 90 days	2 / 24 (8.3%)	4 / 31 (12.9%)	0.71 (0.11–4.45)	0.719	1.34 (0.03–61.30)	0.881

Abbreviations: GERD, Gastroesophageal reflux disease; pts, Patients; 95% CI, 95% confidence interval; PPI, Proton pump inhibitor.

* Significance at $P < 0.05$ †Adjusted for age, sex, income, region of residence, systolic blood pressure, diastolic blood pressure, fasting blood glucose, total cholesterol, obesity, smoking, alcohol consumption, Charlson Comorbidity Index scores, GERD, and H_2 receptor antagonist

Table S5. Subgroup analyses of crude and overlap propensity score weighted odd ratios of H2RA (ref: non user) for mortality in Esophageal cancer participants

Characteristics	Dead participants	Survived participants	Odd ratios for mortality (95% confidence interval)			
	(exposure / total, %)	(exposure / total, %)	Crude	P-value	Overlap weighted model †	P-value
Age < 70 years old (n = 485)						
Exposure history						
Current H2RA	134 / 256 (52.3%)	89 / 229 (38.9%)	1.46 (0.96–2.24)	0.080	1.28 (0.82-1.99)	0.276
Past H2RA	51 / 256 (19.9%)	71 / 229 (31.0%)	0.70 (0.43–1.14)	0.150	0.76 (0.45-1.28)	0.308
Duration of H2RA use						
< 30 days	115 / 256 (44.9%)	104 / 229 (45.4%)	1.07 (0.70–1.64)	0.740	1.01 (0.65-1.57)	0.968
30 to 90 days	41 / 256 (16.0%)	33 / 229 (14.4%)	1.21 (0.69–2.13)	0.514	1.05 (0.58-1.90)	0.879
≥ 90 days	29 / 256 (11.3%)	23 / 229 (10.0%)	1.23 (0.65–2.32)	0.534	1.59 (0.79-3.19)	0.191
Age ≥ 70 years old (n = 326)						
Exposure history						
Current H2RA	123 / 214 (57.5%)	41 / 112 (36.6%)	1.80 (0.97–3.34)	0.062	2.26 (1.17-4.36)	0.016*
Past H2RA	51 / 214 (23.8%)	47 / 112 (42.0%)	0.65 (0.34–1.24)	0.191	0.62 (0.31-1.23)	0.170
Duration of H2RA use						
< 30 days	76 / 214 (35.5%)	49 / 112 (43.8%)	0.93 (0.50–1.73)	0.820	1.05 (0.55-2.00)	0.888
30 to 90 days	53 / 214 (24.8%)	17 / 112 (15.2%)	1.87 (0.89–3.94)	0.099	2.13 (0.98-4.64)	0.056
≥ 90 days	45 / 214 (21.0%)	22 / 112 (19.6%)	1.23 (0.60–2.52)	0.576	1.29 (0.60-2.79)	0.512
Male (n = 756)						
Exposure history						
Current H2RA	244 / 446 (54.7%)	119 / 310 (38.4%)	1.70 (1.19–2.43)	0.004*	1.55 (1.07-2.23)	0.020*
Past H2RA	97 / 446 (21.7%)	104 / 310 (33.5%)	0.77 (0.52–1.15)	0.203	0.65 (0.43-0.98)	0.042*
Duration of H2RA use						
< 30 days	186 / 446 (41.7%)	140 / 310 (45.2%)	1.10 (0.77–1.58)	0.600	1.01 (0.70-1.45)	0.977
30 to 90 days	87 / 446 (19.5%)	41 / 310 (13.2%)	1.76 (1.10–2.81)	0.018*	1.54 (0.96-2.48)	0.076
≥ 90 days	68 / 446 (15.2%)	42 / 310 (13.5%)	1.34 (0.83–2.16)	0.229	1.29 (0.78-2.13)	0.318
Female (n = 55)						
Exposure history						
Current H2RA	13 / 24 (54.2%)	11 / 31 (35.5%)	1.18 (0.30–4.73)	0.814	1.67 (0.05-52.72)	0.772
Past H2RA	5 / 24 (20.8%)	14 / 31 (45.2%)	0.36 (0.08–1.64)	0.186	0.13 (0.00-5.58)	0.291
Duration of H2RA use						
< 30 days	5 / 24 (20.8%)	13 / 31 (41.9%)	0.38 (0.08–1.78)	0.221	0.33 (0.01-10.98)	0.537
30 to 90 days	7 / 24 (29.2%)	9 / 31 (29.0%)	0.78 (0.17–3.49)	0.743	0.36 (0.01-12.91)	0.579
≥ 90 days	6 / 24 (25.0%)	3 / 31 (9.7%)	2.00 (0.33–11.97)	0.448	1.47 (0.02-95.92)	0.857

Abbreviations: GERD, Gastroesophageal reflux disease; pts, Patients; 95% CI, 95% confidence interval; H2RA, H_2 receptor antagonist.

* Significance at $P < 0.05$ †Adjusted for age, sex, income, region of residence, systolic blood pressure, diastolic blood pressure, fasting blood glucose, total cholesterol, obesity, smoking, alcohol consumption, Charlson Comorbidity Index scores, GERD, and proton pump inhibitor.
